# Relative Device-Output Music Intensity and Virtual-Reality-Based Postural Control in Trained Athletes

**DOI:** 10.3390/brainsci16080772

**Published:** 2026-07-23

**Authors:** Hanifi Korkmaz, İpek Balıkçı Çiçek, Özgür Eken, Monira I. Aldhahi

**Affiliations:** 1 Battalgazi Vocational School, Malatya Turgut Ozal University, Battalgazi, Malatya 44280, Türkiye; hanifi.korkmaz@ozal.edu.tr; 2 Department of Biostatistics and Medical Informatics, Faculty of Medicine, Inonu University, Battalgazi, Malatya 44280, Türkiye; ipek.balikci@inonu.edu.tr; 3 Department of Physical Education and Sport Teaching, Faculty of Sport Sciences, Inonu University, Battalgazi, Malatya 44280, Türkiye; ozgur.eken@inonu.edu.tr; 4 Department of Rehabilitation Sciences, College of Health and Rehabilitation Sciences, Princess Nourah bint Abdulrahman University, P.O. Box 84428, Riyadh 11671, Saudi Arabia

**Keywords:** postural control, relative music intensity, auditory stimulation, virtual reality, CTSIB, limits of stability, trained athletes, sensory reweighting

## Abstract

**Highlights:**

**What are the main findings?**

- Relative device-output music intensity altered outcomes of the Clinical Test of Sensory Interaction in Balance delivered through virtual reality (CTSIB-VR) more consistently than Limits of Stability (LOS) outcomes.
- Sport-related response patterns were exploratory because the sport subgroups were small and unequal.

**What are the implications of the main findings?**

- Auditory stimulation should be treated as an experimental factor rather than a neutral background feature during VR-based balance assessment.
- Future studies should calibrate ear-level sound pressure and confirm sport-stratified CTSIB-VR and LOS patterns in larger, balanced samples.

**Abstract:**

Background/Objectives: Postural control depends on the integration and reweighting of visual, somatosensory, vestibular, and contextual sensory information. Stable auditory cues may support balance, whereas complex musical stimulation may impose additional sensory-cognitive demand during multisensory conflict. This study examined the acute effects of relative device-output music intensity on virtual-reality-based postural control in trained athletes and explored whether responses differed by sport background. Methods: Forty-eight athletes from tennis, combat sports, swimming, football, and volleyball completed the Clinical Test of Sensory Interaction in Balance delivered through virtual reality (CTSIB-VR) and Limits of Stability (LOS) assessments under four auditory conditions: routine/no sound and low (+10 dB), moderate (+20 dB), and high (+30 dB) relative device-output increments. Linear mixed-effects models included sport, auditory condition, and their interaction as fixed effects and participant-specific random intercepts and random linear condition slopes. Model-based estimated marginal means, Bonferroni-adjusted contrasts, 1.5×IQR sensitivity analyses, and robust generalized estimating equations were calculated. Results: Auditory condition affected all five CTSIB-VR outcomes (Wald χ^2^(3) = 16.773–94.404, all *p* < 0.001). The routine condition exceeded the high-intensity condition for composite score (adjusted mean difference = 6.05, 95% CI 3.99–8.10; Bonferroni-adjusted *p* < 0.001) and somatosensory score (8.62, 95% CI 6.78–10.46; adjusted *p* < 0.001). Sport × condition interactions were significant for all CTSIB-VR outcomes (χ^2^(12) = 54.869–98.953, all *p* < 0.001), but sport-stratified findings were exploratory. For LOS, auditory-condition effects were detected for endpoint excursion (*p* = 0.004), maximum excursion (*p* < 0.001), and directional control (*p* = 0.002), whereas reaction time (*p* = 0.648) and movement velocity (*p* = 0.056) did not show clear main effects. Sensitivity analyses supported the endpoint-excursion, maximum-excursion, and directional-control findings; movement-velocity inference was method-sensitive. Conclusions: Relative device-output music intensity was associated with consistent changes in CTSIB-VR sensory-organization measures and outcome-specific changes in LOS performance. Sport-related patterns require confirmation in adequately powered, balanced samples.

## 1. Introduction

Postural control is an active sensorimotor process through which the body maintains or restores equilibrium during quiet stance and movement. This process depends on the continuous integration and reweighting of visual, somatosensory, and vestibular information according to the reliability of each sensory channel and the demands of the task [1,2,3,4]. When one source of information becomes inaccurate or unavailable, the central nervous system must adaptively prioritize other sensory inputs to preserve postural orientation and stability [2,3].

Although visual, vestibular, and somatosensory contributions to balance have been widely examined, auditory input has received comparatively less attention. Evidence regarding auditory contributions is heterogeneous. Stable spatialized sounds and low-level auditory noise can reduce sway by providing external reference information or enhancing sensory detection [5,6,7,8,9,10,11,12], whereas music or nonspatial acoustic stimulation has produced context-dependent, null, or destabilizing responses [10,13,14,15]. Thus, sound is not a unitary intervention: spatial informativeness, spectral structure, intensity, task difficulty, and attentional demand may determine whether auditory input supports or competes with postural control.

Music represents a particularly complex auditory stimulus. Unlike simple tones or spatial environmental sounds, music contains rhythmic, melodic, affective, and arousal-related components that may influence attention and motor control [16,17]. These characteristics make music relevant to sport and rehabilitation settings, but they also complicate interpretation. Depending on task demands and stimulus properties, music may either support movement regulation or interfere with postural control by increasing attentional load. Sound intensity may further influence this response; however, few studies have examined whether increasing music intensity alters balance performance under controlled multisensory conflict.

Virtual-reality-based balance assessment provides a useful framework for studying these effects because it allows standardized manipulation of sensory conditions while preserving a more immersive perceptual environment than conventional static balance tasks [18,19,20]. In this context, the Clinical Test of Sensory Interaction in Balance delivered through virtual reality provides performance-based information about sensory organization during stance, whereas the Limits of Stability test evaluates voluntary control of the center of gravity near the boundaries of stability [21,22]. Examining both domains is important because auditory stimulation may affect constrained sensory organization and voluntary postural control differently.

Athletes are a relevant population because long-term sport participation may shape sensory-weighting strategies. Potential differences among the included sports were considered exploratory rather than confirmatory. Tennis and volleyball require continuous visual tracking and anticipatory responses to a moving ball; football combines open-skill visuospatial monitoring with rapid whole-body redirection; combat sports emphasize close-range anticipatory control and rapid proprioceptive and vestibular adjustments; and swimming is performed in an environment with altered visual and somatosensory cues. These differing long-term exposures may be associated with distinct sensory-control strategies [23,24,25]. However, baseline differences may also contribute to apparent sport × condition interactions, and the present subgroup sizes are insufficient to establish sport-specific mechanisms.

Therefore, this study examined the acute effects of relative device-output music intensity on CTSIB-VR and LOS outcomes in trained athletes from different sport backgrounds. We hypothesized that auditory condition would influence postural-control outcomes, that these effects would differ by sport type, and that auditory effects would be more pronounced for CTSIB-VR sensory-organization outcomes than for LOS outcomes. Because sound exposure was standardized using device-output increments rather than independently calibrated ear-level sound pressure levels, the study was framed as an investigation of relative music intensity rather than absolute acoustic dose.

## 2. Materials and Methods

### 2.1. Study Design and Participants

This study used a within-subject repeated-measures experimental design. The analytic sample comprised 48 trained athletes aged 18–30 years: tennis (*n* = 14), combat sports (*n* = 11), swimming (*n* = 9), football (*n* = 8), and volleyball (*n* = 6). One additional gymnast record was not included because gymnastics was not among the five prespecified sport strata and the low-intensity condition was absent. Participants were classified as trained athletes if they had at least five years of regular participation in organized sport. Inclusion criteria were age of 18–30 years, at least five years of organized sport participation, normal bilateral hearing thresholds defined as a pure-tone average of ≤20 dB HL at 500, 1000, 2000, and 4000 Hz, and written informed consent. Exclusion criteria were self-reported or clinically identified hearing loss, active ear pathology, vestibular disorder or vertigo, neurological or psychiatric diagnosis, an orthopedic condition affecting balance, or inability to complete the protocol. No formal a priori sample-size calculation was performed. Because the sport subgroups were small and unequal, all sport-specific contrasts and sport × condition interactions were treated as exploratory and hypothesis-generating.

### 2.2. Ethics

The study was approved by the Health Sciences Ethics Committee of Malatya Turgut Özal University (decision no. 2025/95). All procedures were conducted in accordance with the Declaration of Helsinki. Written informed consent was obtained from all participants before testing.

### 2.3. Auditory Stimulation Protocol

Musical stimulation was delivered through the integrated headphones of the Virtualis Balance VR system (Virtualis, Pérols, France). All participants listened to the same standardized instrumental excerpt from Mozart’s Jupiter Symphony. Four auditory conditions were administered in randomized order: routine/no sound, low (+10 dB), moderate (+20 dB), and high (+30 dB) device-output increments. The same playback device and headphones were used for all participants and conditions. The archived records available for this revision did not preserve the randomization algorithm, whether sequences were generated independently for each participant, or a counterbalancing matrix; therefore, the precise allocation procedure and complete counterbalancing cannot be verified and are not claimed. A seated rest interval of at least 60 s was provided between conditions to reduce immediate carry-over, although residual order, learning, fatigue, or carry-over effects cannot be excluded. Sound pressure level was not independently measured at the ear canal; the conditions therefore represent standardized relative device-output settings rather than calibrated absolute sound-pressure-level exposures.

### 2.4. Virtual-Reality-Based CTSIB Assessment

Postural control was assessed using the Clinical Test of Sensory Interaction in Balance implemented in the Virtualis Balance VR platform (Virtualis, Pérols, France) [26]. Under each auditory condition, participants completed six sensory conditions: firm surface/eyes open, firm surface/eyes closed, firm surface with sway-referenced visual surround, foam surface/eyes open, foam surface/eyes closed, and foam surface with sway-referenced visual surround. Each trial lasted 20 s and was repeated three times. The primary CTSIB outcomes were composite, somatosensory, visual, vestibular, and visual preference scores, with higher values indicating better performance. These outcomes were interpreted as performance-based indicators of sensory organization rather than direct neurophysiological measures of sensorimotor integration.

### 2.5. Limits of Stability Assessment

LOS outcomes were obtained under the same auditory conditions using the Virtualis Balance VR platform. The software-derived outcomes were reaction time, movement velocity, endpoint excursion, maximum excursion, and directional control. Lower reaction time indicates faster response initiation; higher values for the other measures generally indicate better performance, subject to movement strategy and data quality. The archived study documentation available for this revision did not specify the number or directions of trials, the exact instructions provided, familiarization procedures, or formal trial-repetition and validity criteria. These details cannot be reconstructed reliably and their absence is acknowledged as a limitation of procedural reproducibility.

### 2.6. Noise Sensitivity Assessment

Subjective noise sensitivity was assessed before postural testing using the validated Turkish version of the Weinstein Noise Sensitivity Scale [27,28]. WNSS was treated as a descriptive/exploratory participant characteristic rather than as a primary mechanistic endpoint because the study was not powered for a prespecified moderator model across all outcomes. Future studies should test whether noise sensitivity or sensory processing sensitivity modifies the postural response to auditory stimulation [27,28,29].

### 2.7. Statistical Analysis

Descriptive results are reported as mean ± standard deviation, with cell-level 95% confidence intervals provided in Supplementary File S1. For each outcome, a linear mixed-effects model included auditory condition (four categorical levels), sport type (five levels), and their interaction as fixed effects. Sum-to-zero contrasts were used. Participant-specific random intercepts and random slopes for a standardized ordinal condition score (0 = routine/no sound, 1 = low, 2 = moderate, 3 = high) modeled within-participant dependence without imposing a linear fixed effect of intensity. Maximum-likelihood comparisons showed that this random-slope structure improved model fit over random-intercept-only models for every outcome (likelihood-ratio *p* ≤ 0.004); final estimates were obtained by restricted maximum likelihood. Fixed-effect blocks were evaluated using joint Wald χ^2^ tests. Equal-weight estimated marginal means were calculated, and pairwise auditory-condition contrasts, within-sport condition contrasts, and between-sport contrasts within each condition were Bonferroni-adjusted within their respective comparison families. All available outcome observations were analyzed without imputation; one CTSIB visual value and one high-condition LOS record for movement velocity, endpoint excursion, maximum excursion, and directional control were missing. Model diagnostics included convergence, residual-versus-fitted and quantile–quantile inspection, residual skewness and kurtosis, and evaluation of the random-effects covariance matrix. For LOS outcomes, sensitivity analyses excluded observations outside the 1.5 × interquartile-range fences and separately used Gaussian generalized estimating equations with exchangeable working correlation and robust sandwich standard errors. Analyses were conducted in Python 3.13 using SciPy 1.17.0, statsmodels 0.14.6, and patsy 1.0.2; two-sided *p* < 0.05 indicated statistical significance.

## 3. Results

Auditory condition affected all five CTSIB-VR outcomes, whereas LOS effects were outcome-specific. The primary mixed-effects-model results are summarized in Table 1. Complete fixed-effect coefficients, estimated marginal means, corrected pairwise contrasts, model-structure comparisons, diagnostics, sensitivity analyses, and the de-identified participant-level condition dataset are provided in Supplementary File S1.

Interpretation. Auditory condition and sport × condition interaction effects were present across all CTSIB-VR outcomes. For LOS, endpoint excursion, maximum excursion, and directional control showed condition and interaction effects that were retained in sensitivity analyses; reaction time showed an interaction without a condition main effect, and movement velocity was method-sensitive. Sport-stratified findings remain exploratory.

### 3.1. CTSIB Outcomes

CTSIB composite score showed effects of sport type (χ^2^(4) = 19.318, *p* < 0.001), auditory condition (χ^2^(3) = 36.995, *p* < 0.001), and sport × condition interaction (χ^2^(12) = 98.953, *p* < 0.001). The routine estimated marginal mean exceeded low (difference = 4.33, 95% CI 2.41–6.26), moderate (3.82, 95% CI 1.73–5.91), and high (6.05, 95% CI 3.99–8.10) conditions after Bonferroni correction. Within-sport contrasts were concentrated in tennis, where routine exceeded all music conditions and high was lower than low and moderate; these subgroup results are exploratory.

Table 2 shows generally higher CTSIB composite scores in the routine/no-sound condition. No corrected sport contrast was significant in the routine condition, whereas tennis differed from several sports during the music conditions, indicating divergence after auditory stimulation rather than a uniformly lower baseline. Because subgroup sizes were small and unequal, this pattern remains hypothesis-generating.

**Figure 1:**
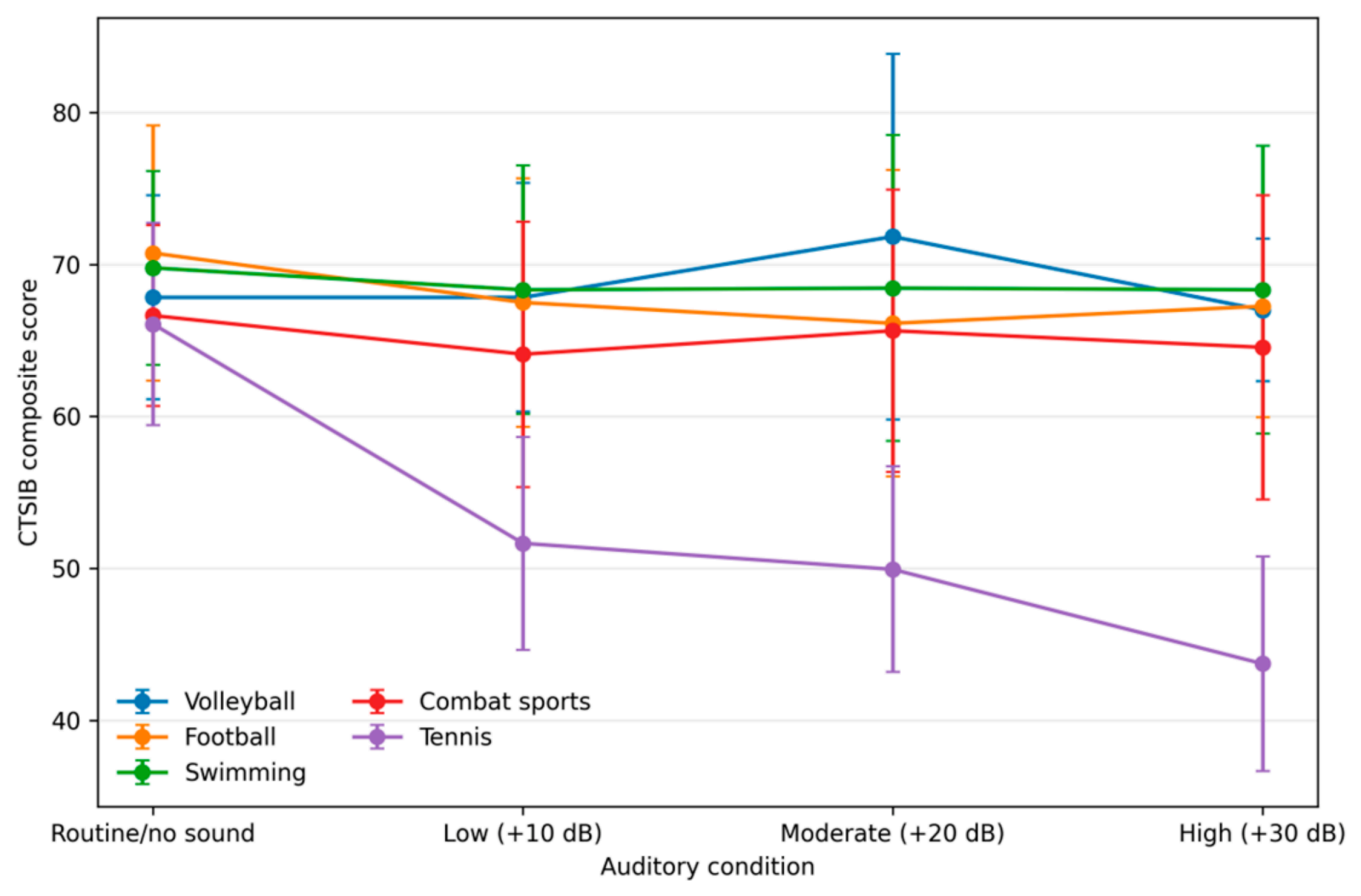
CTSIB composite score across auditory conditions by sport type. Points represent means and error bars represent 95% confidence intervals.

Figure 1 shows mean CTSIB composite scores with 95% confidence intervals. The sport-specific trajectories are descriptive and should not be interpreted as confirmatory subgroup effects.

As shown in Table 3, somatosensory score showed an auditory-condition effect (χ^2^(3) = 94.404, *p* < 0.001) and sport × condition interaction (χ^2^(12) = 80.710, *p* < 0.001), without a sport main effect (*p* = 0.356). The routine estimated marginal mean exceeded low, moderate, and high conditions, and the high condition was lower than low and moderate after correction. Significant exploratory within-sport contrasts were most numerous in tennis, with additional routine-versus-high or routine-versus-low differences in football and swimming.

**Figure 2:**
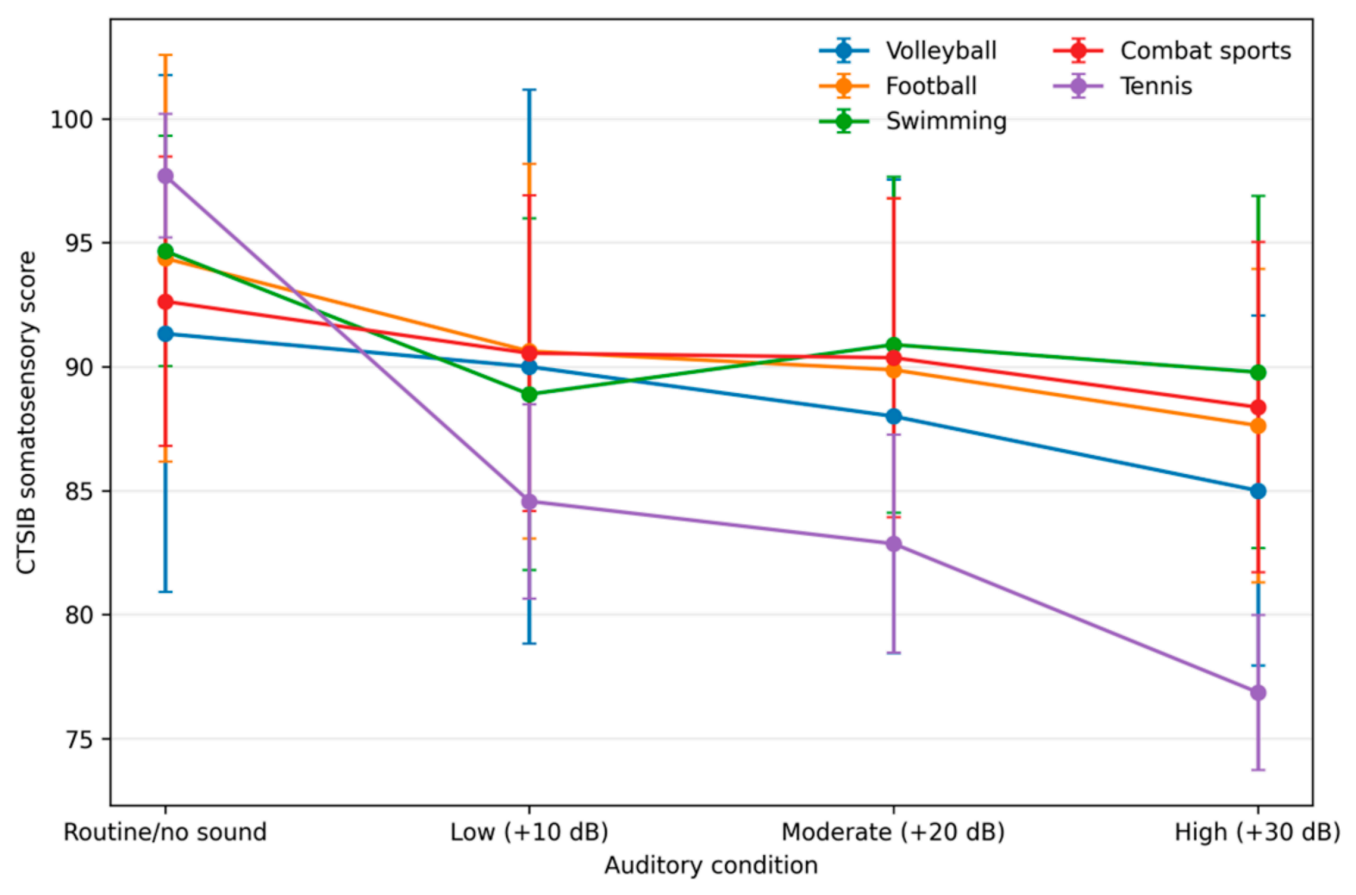
CTSIB somatosensory score across auditory conditions by sport type. Points represent means and error bars represent 95% confidence intervals.

Figure 2 shows mean somatosensory scores with 95% confidence intervals. The larger tennis decline is an exploratory pattern and does not establish a tennis-specific mechanism.

In Table 4, visual score showed effects of sport type (χ^2^(4) = 37.583, *p* < 0.001), auditory condition (χ^2^(3) = 16.773, *p* < 0.001), and interaction (χ^2^(12) = 96.560, *p* < 0.001). Routine exceeded low and moderate in the overall estimated-marginal-mean contrasts. Tennis had lower visual scores than several sports even in the routine condition, and routine exceeded all three music conditions within tennis; therefore, both pre-existing baseline differences and condition-related divergence contributed to the interaction.

**Figure 3:**
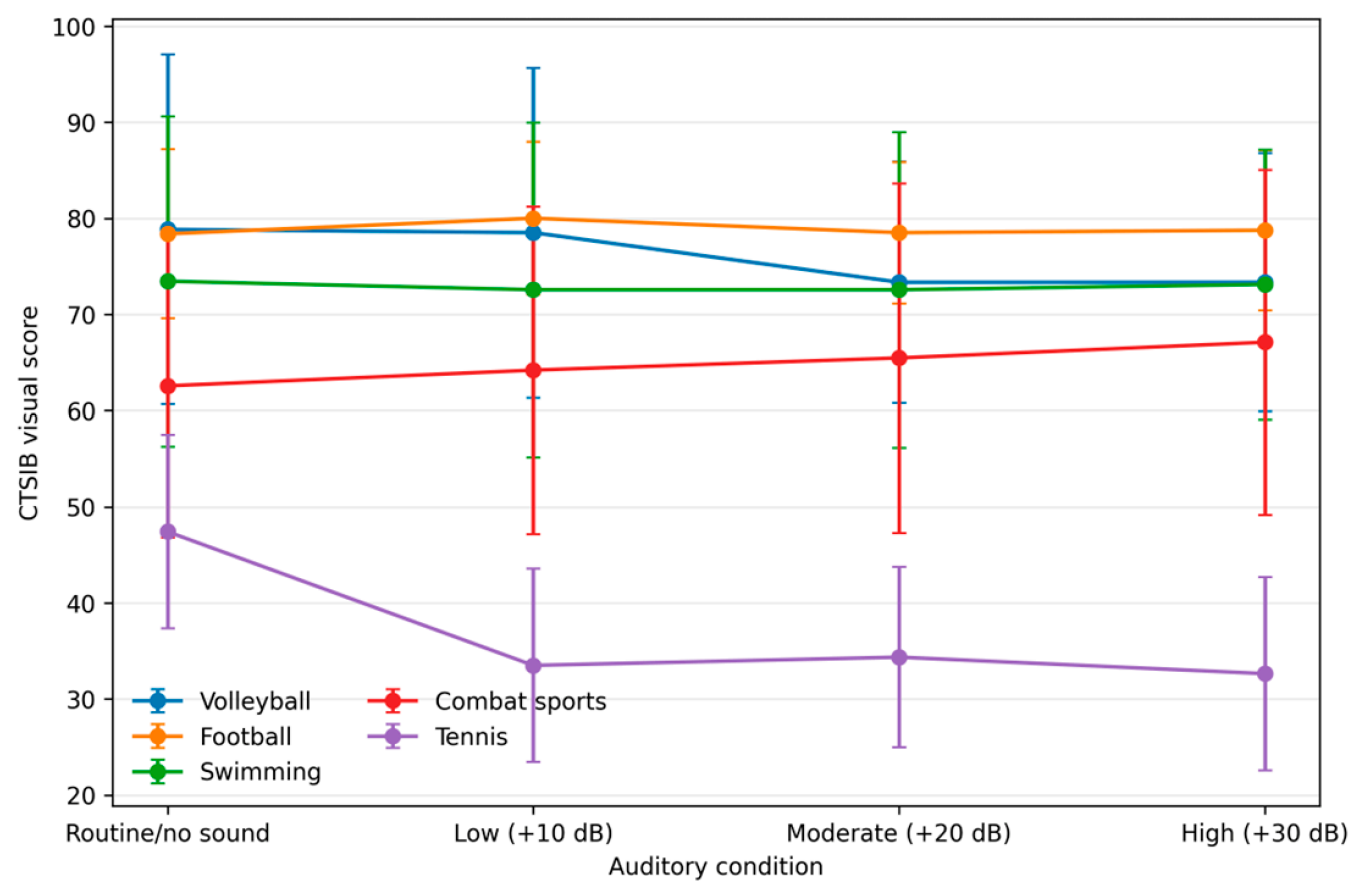
CTSIB visual score across auditory conditions by sport type. Points represent means and error bars represent 95% confidence intervals.

Figure 3 displays mean visual CTSIB-VR scores with 95% confidence intervals. Baseline differences and unequal subgroup sizes require cautious interpretation.

In Table 5, the vestibular score showed effects of sport type (χ^2^(4) = 23.170, *p* < 0.001), auditory condition (χ^2^(3) = 30.253, *p* < 0.001), and interaction (χ^2^(12) = 54.869, *p* < 0.001). Routine exceeded all music conditions in the overall contrasts. Within tennis, routine exceeded low, moderate, and high, and moderate exceeded high after correction; these findings remain exploratory.

**Figure 4:**
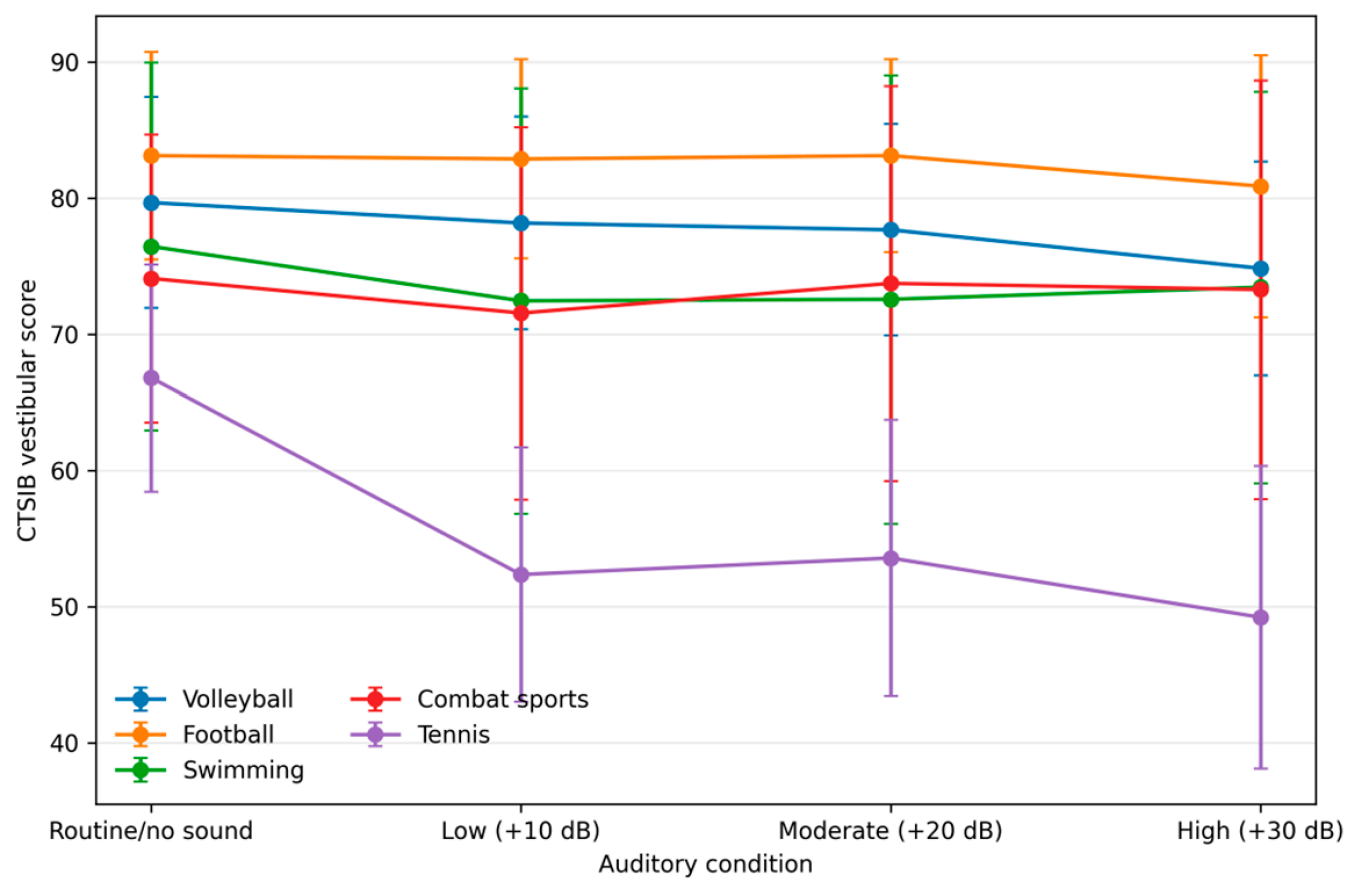
CTSIB vestibular score across auditory conditions by sport type. Points represent means and error bars represent 95% confidence intervals.

Figure 4 shows mean vestibular scores with 95% confidence intervals. The observed tennis trajectory should not be interpreted as a confirmed sport-specific effect.

In Table 6, the visual-preference score showed effects of sport type (χ^2^(4) = 27.768, *p* < 0.001), auditory condition (χ^2^(3) = 26.368, *p* < 0.001), and interaction (χ^2^(12) = 70.015, *p* < 0.001). Routine exceeded all music conditions in the overall contrasts. The largest corrected within-sport contrasts occurred in tennis, including routine versus all music conditions and low or moderate versus high; these are exploratory subgroup findings.

**Figure 5:**
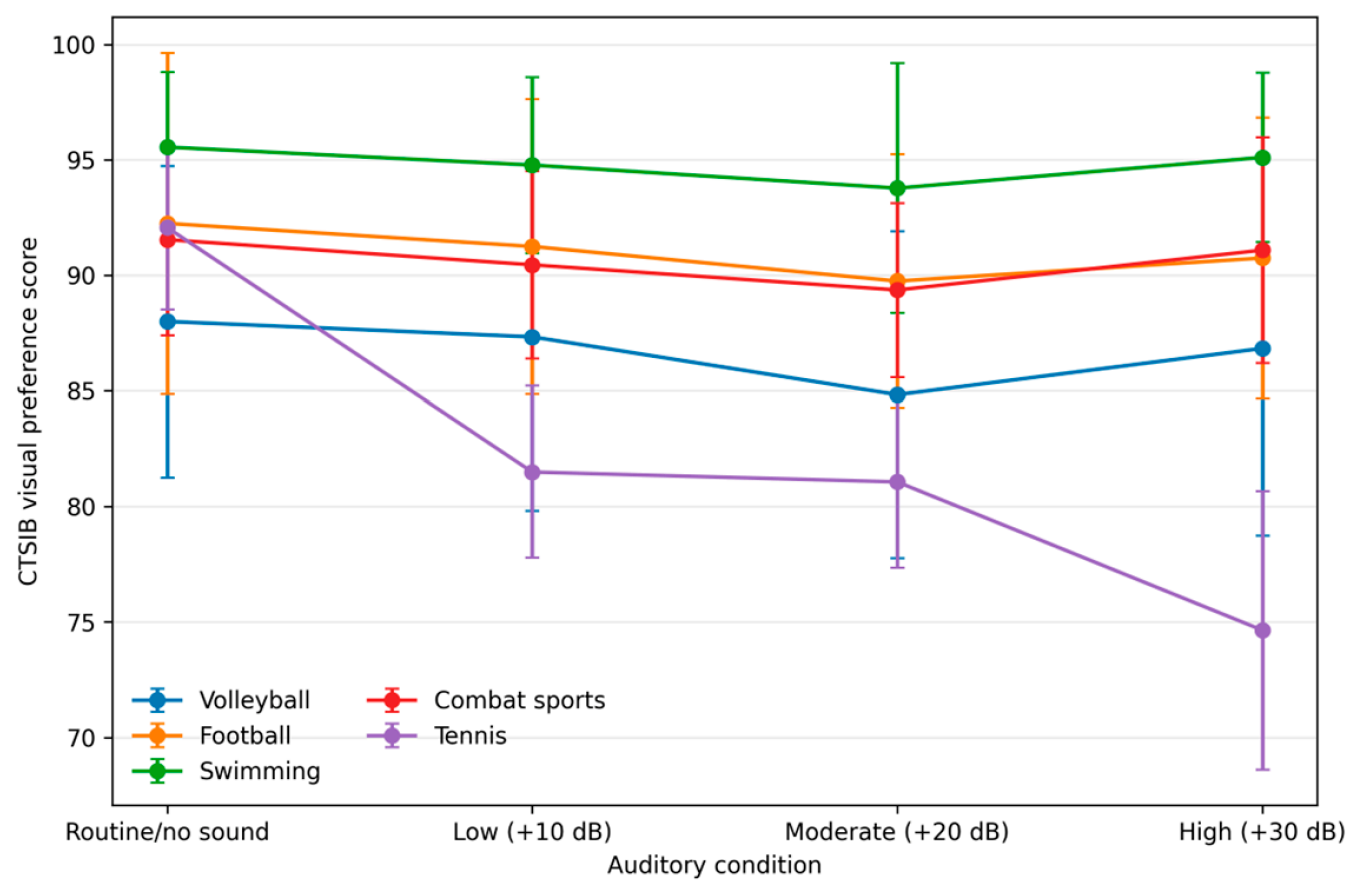
CTSIB visual preference score across auditory conditions by sport type. Points represent means and error bars represent 95% confidence intervals.

Figure 5 shows mean visual-preference scores with 95% confidence intervals. Sport-specific trajectories remain hypothesis-generating.

### 3.2. LOS Outcomes

LOS outcomes were less uniform than CTSIB-VR outcomes. Reaction time showed no auditory-condition main effect (χ^2^(3) = 1.652, *p* = 0.648) but showed a sport × condition interaction (χ^2^(12) = 25.428, *p* = 0.013). Movement velocity had neither a conventionally significant condition effect (χ^2^(3) = 7.571, *p* = 0.056) nor interaction (*p* = 0.357), and sensitivity results were method-dependent. Endpoint excursion showed condition (χ^2^(3) = 13.508, *p* = 0.004) and interaction effects (χ^2^(12) = 65.304, *p* < 0.001). Maximum excursion also showed condition (χ^2^(3) = 22.718, *p* < 0.001) and interaction effects (χ^2^(12) = 65.737, *p* < 0.001). Directional control showed sport (χ^2^(4) = 13.025, *p* = 0.011), condition (χ^2^(3) = 15.204, *p* = 0.002), and interaction effects (χ^2^(12) = 65.947, *p* < 0.001). One tennis high-condition value was missing for movement velocity, endpoint excursion, maximum excursion, and directional control.

As shown in Table 7, the reaction time did not show a condition main effect. Within tennis, high-intensity reaction time was slower than routine, low, and moderate after correction, whereas volleyball showed the opposite descriptive direction. The interaction remained significant after IQR exclusion and in robust GEE analysis, but the sport-stratified contrasts are exploratory.

**Figure 6:**
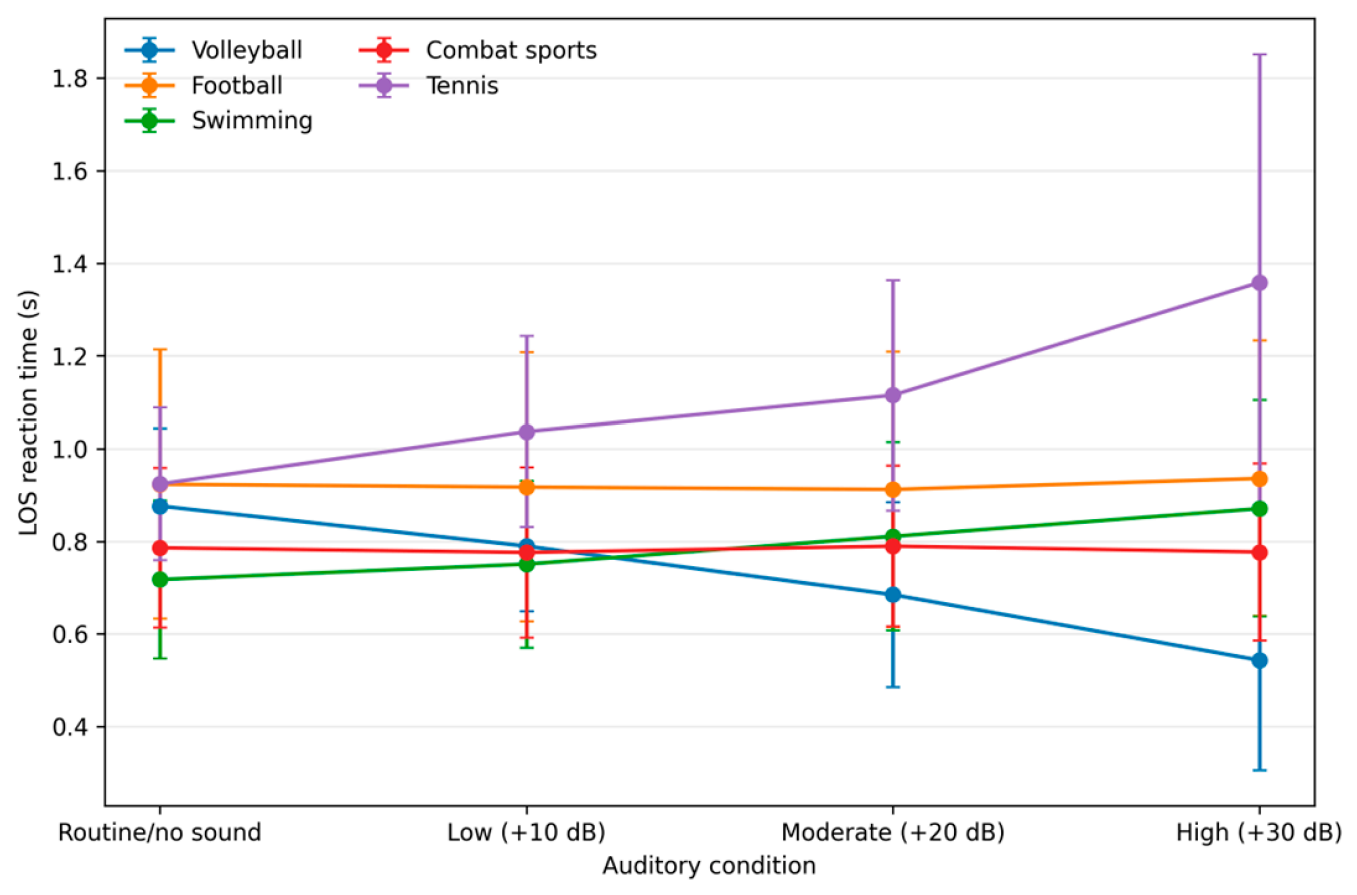
LOS reaction time across auditory conditions by sport type. Points represent means and error bars represent 95% confidence intervals.

Figure 6 shows all available subgroup means with 95% confidence intervals. All observed reaction-time values were retained in the primary analysis.

As shown in Table 8, the movement-velocity condition effect was near the conventional threshold in the primary model (*p* = 0.056), significant after IQR exclusion (*p* = 0.031), and significant in robust GEE analysis (*p* < 0.001). Because the inferential conclusion depended on analytic specification, movement velocity was treated as method-sensitive rather than as a definitive auditory-condition effect.

**Figure 7:**
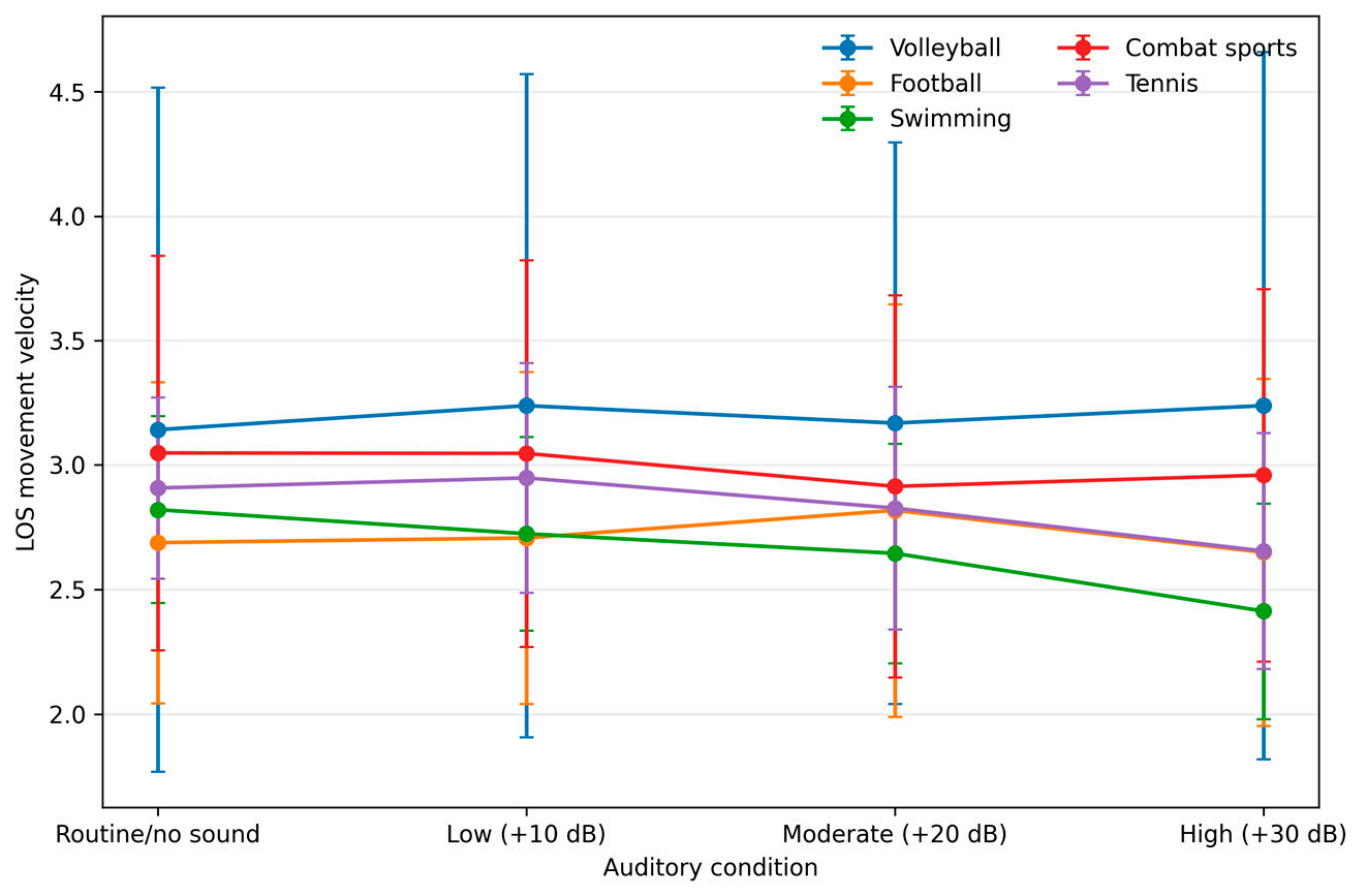
LOS movement velocity across auditory conditions by sport type. Points represent means and error bars represent 95% confidence intervals.

Figure 7 shows mean movement velocity with 95% confidence intervals. Apparent subgroup differences are descriptive.

In Table 9, the endpoint excursion showed condition and interaction effects. Overall, routine and low estimated marginal means exceeded high after correction. Exploratory within-sport contrasts indicated reductions across intensity in tennis and swimming, smaller high-condition values in football, and an opposite high-condition increase in volleyball. The condition and interaction effects remained significant after IQR exclusion and in robust GEE analysis.

**Figure 8:**
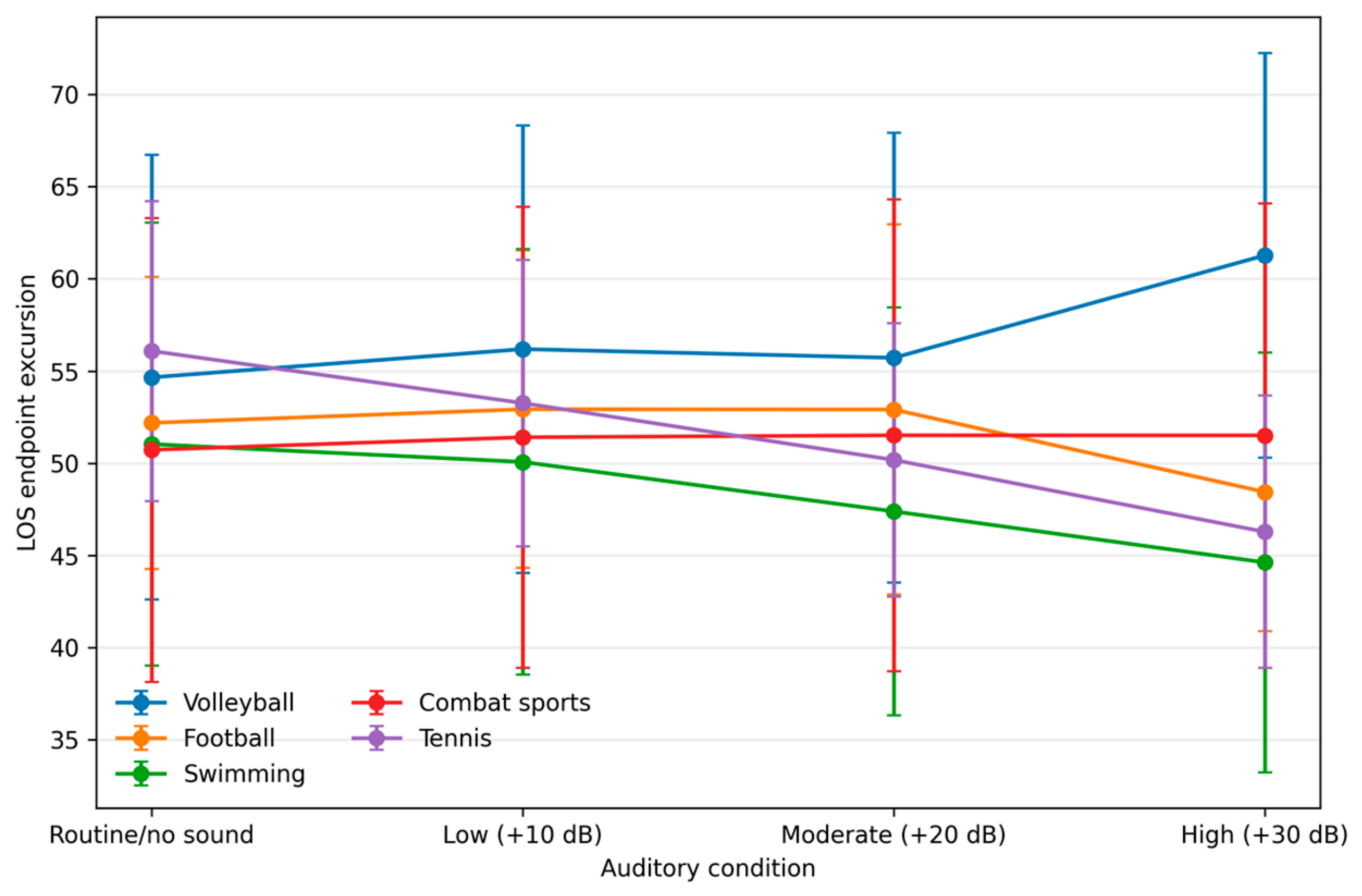
LOS endpoint excursion across auditory conditions by sport type. Points represent means and error bars represent 95% confidence intervals.

Figure 8 shows mean endpoint excursion with 95% confidence intervals. Opposing subgroup trajectories explain why the interaction should not be summarized as a uniform effect.

In Table 10, the maximum excursion showed condition and interaction effects. Routine exceeded moderate and high, and low exceeded high in the overall corrected contrasts. Exploratory simple effects showed progressive reductions in tennis and swimming, whereas volleyball displayed comparatively stable or higher values at greater intensity. Both omnibus effects remained significant in IQR-excluded and robust GEE sensitivity analyses.

**Figure 9:**
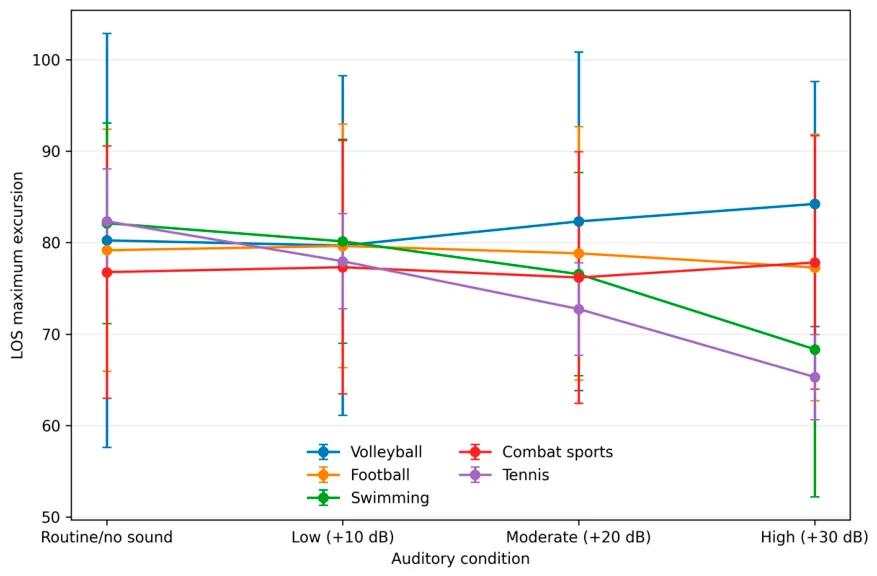
LOS maximum excursion across auditory conditions by sport type. Points represent means and error bars represent 95% confidence intervals.

Figure 9 shows mean maximum excursion with 95% confidence intervals. Subgroup directions are exploratory.

Directional control showed sport, condition, and interaction effects. Overall, routine exceeded high; low exceeded moderate and high; and moderate exceeded high after correction. Exploratory contrasts showed progressive reductions in tennis and swimming and higher values at greater intensity in volleyball. The condition and interaction effects were retained in robust GEE analysis (Table 11).

**Figure 10:**
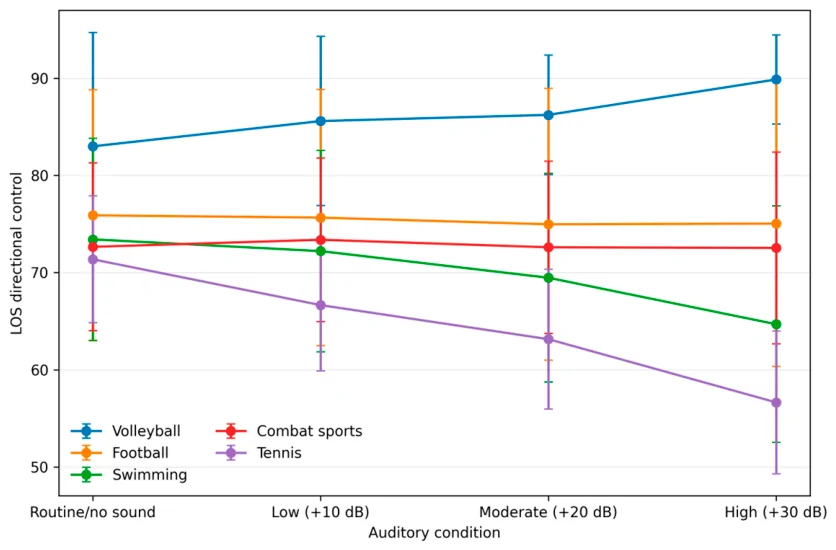
LOS directional control across auditory conditions by sport type. Points represent means and error bars represent 95% confidence intervals.

Figure 10 shows all available subgroup means with 95% confidence intervals. The tennis high-intensity estimate was calculated from the available observations because one directional-control value was missing.

### 3.3. Sensitivity Analyses and Statistical Robustness

The 1.5 × IQR rule flagged 12 reaction-time observations, 10 movement-velocity observations, four endpoint-excursion observations, five maximum-excursion observations, and no directional-control observations. For reaction time, the condition main effect remained non-significant, while the interaction remained significant after IQR exclusion and in robust GEE analysis; the sport main effect was not stable. Movement velocity was method-sensitive, with a non-significant primary condition test but significant IQR-excluded and robust-GEE tests. Endpoint excursion, maximum excursion, and directional control retained their principal condition and interaction findings across sensitivity analyses. Complete flagged-observation lists and all sensitivity-model outputs are provided in Supplementary File S1.

## 4. Discussion

This study examined whether relative device-output music intensity was associated with acute changes in VR-based postural-control outcomes in trained athletes. The most consistent evidence concerned CTSIB-VR: all five sensory-organization outcomes showed auditory-condition effects and sport × condition heterogeneity. LOS results were more outcome-specific. Endpoint excursion, maximum excursion, and directional control showed condition and interaction effects that were supported by sensitivity analyses; reaction time showed an exploratory interaction without a condition main effect, and movement velocity was method-sensitive.

The CTSIB-VR findings are consistent with a heterogeneous auditory-balance literature. Stable spatial or environmental sounds and auditory noise may improve stability by supplying an external reference or enhancing sensory detection [5,6,7,8,9,10,11,12], whereas music and other nonspatial stimuli can produce context-dependent, null, or destabilizing effects [10,13,14,15]. The present musical excerpt, delivered through integrated VR headphones, provided limited external spatial anchoring while introducing rhythmic, melodic, affective, and attentional content [16,17]. Under multisensory conflict, that content may have competed with sensory organization. Thus, the findings should not be generalized to sound as a whole; they apply to the relative device-output musical conditions tested here.

Sport × condition interactions indicated heterogeneous trajectories, but the sport-stratified findings remain exploratory. Tennis showed the largest observed reductions in several CTSIB-VR measures. For visual score, tennis was already lower than several sports in the routine condition, demonstrating that baseline differences contributed to the interaction. For composite score, corrected sport differences were absent at routine but emerged during music conditions, suggesting divergence after stimulation. These patterns are compatible with sport-related sensory-control differences [23,24,25], but they may also reflect sampling variability, unequal group sizes, unmeasured training characteristics, or regression to the mean. They should not be interpreted as evidence that tennis participation causes greater auditory susceptibility.

The LOS findings demonstrate that auditory effects depended on the control domain. CTSIB-VR emphasizes maintenance of stance under altered sensory conditions, whereas LOS requires intentional displacement toward stability boundaries. Endpoint excursion, maximum excursion, and directional control changed across auditory conditions, but subgroup directions were not uniform: tennis and swimming often declined, whereas volleyball sometimes increased. Reaction time showed opposing sport trajectories without an overall condition effect, and movement velocity depended on analytic specification. Thus, the data do not support a single beneficial or detrimental effect of music on voluntary stability-limit control.

Sensitivity analyses materially changed the interpretation of the LOS results. The previously suspected directional-control anomaly was a data-entry error and was corrected in the verified dataset; no directional-control observation was flagged by the IQR rule. The reaction-time interaction persisted after exclusion of flagged observations and under robust GEE, although subgroup estimates remained imprecise. Endpoint-excursion, maximum-excursion, and directional-control condition and interaction effects were also retained. In contrast, movement-velocity inference varied across models and was treated as inconclusive.

These findings have methodological implications for VR-based postural assessment. Auditory stimulation should not be treated as a neutral background feature. Stable spatial cues, broadband noise, musical stimulation, and silence differ in spatial informativeness and cognitive-affective content. The type of sound, delivery method, calibrated ear-level intensity, familiarity, preference, perceived loudness, annoyance, affective response, and attentional load may all alter performance. Future studies should measure these variables and prespecify comparisons among music, spatialized environmental sounds, noise, metronomic rhythms, and silence.

Several limitations should be considered. First, the auditory conditions were relative device-output increments rather than independently calibrated ear-level sound-pressure levels, precluding conclusions about absolute acoustic dose. Second, sport subgroups were small and unequal and no a priori sample-size calculation was performed; sport-stratified contrasts and interactions are therefore exploratory. Third, although condition order was described as randomized, the archived records did not preserve the randomization algorithm or a counterbalancing matrix, and detailed familiarization, LOS trial directions, instructions, and formal validity criteria could not be reconstructed; residual order, learning, fatigue, and carry-over effects cannot be excluded. Fourth, perceived loudness, music familiarity, preference, annoyance, affective response, and attentional demand were not measured, and noise sensitivity was not modeled as a prespecified moderator. Fifth, the available dataset contained participant-level condition summaries rather than individual trial-level measurements, limiting trial-level reliability and error modeling. Sixth, the participants were young, trained athletes, who may have sensory-reweighting and compensatory capacities that differ from non-athletes and from people with vestibular dysfunction, post-stroke balance impairment, or high fall risk; clinical and population generalizability is limited. Finally, the single-session acute design does not establish adaptation, training effects, or transfer to sport-specific performance.

## 5. Conclusions

Relative device-output music intensity was associated with acute changes in VR-based postural control. Effects were most consistent across CTSIB-VR sensory-organization measures, while LOS responses were outcome-specific: endpoint excursion, maximum excursion, and directional control were supported by sensitivity analyses, reaction time showed an exploratory interaction without a main condition effect, and movement velocity was method-sensitive. Sport-related patterns, including the larger observed CTSIB-VR reductions in tennis, remain exploratory and require confirmation in adequately powered, balanced studies with calibrated ear-level sound exposure and fully documented randomization and assessment procedures.

## Figures and Tables

**Table 1:** Primary linear mixed-effects-model omnibus results.

Outcome	Sport Type	Auditory Condition	Sport × Auditory Condition	Interpretation
CTSIB composite	χ^2^(4) = 19.318, *p* < 0.001	χ^2^(3) = 36.995, *p* < 0.001	χ^2^(12) = 98.953, *p* < 0.001	Routine exceeded all music conditions; subgroup patterns exploratory.
CTSIB somatosensory	χ^2^(4) = 4.387, *p* = 0.356	χ^2^(3) = 94.404, *p* < 0.001	χ^2^12) = 80.710, *p* < 0.001	Strong condition effect; largest exploratory declines in tennis.
CTSIB visual	χ^2^(4) = 37.583, *p* < 0.001	χ^2^(3) = 16.773, *p* < 0.001	χ^2^(12) = 96.560, *p* < 0.001	Baseline sport differences and condition-related divergence contributed.
CTSIB vestibular	χ^2^(4) = 23.170, *p* < 0.001	χ^2^(3) = 30.253, *p* < 0.001	χ^2^(12) = 54.869, *p* < 0.001	Routine generally higher; subgroup patterns exploratory.
CTSIB visual preference	χ^2^(4) = 27.768, *p* < 0.001	χ^2^(3) = 26.368, *p* < 0.001	χ^2^(12) = 70.015, *p* < 0.001	Routine generally higher; subgroup patterns exploratory.
LOS reaction time	χ^2^(4) = 9.501, *p* = 0.050	χ^2^(3) = 1.652, *p* = 0.648	χ^2^(12) = 25.428, *p* = 0.013	No condition main effect; exploratory interaction retained in sensitivity analyses.
LOS movement velocity	χ^2^(4) = 1.848, *p* = 0.764	χ^2^(3) = 7.571, *p* = 0.056	χ^2^(12) = 13.166, *p* = 0.357	Near-threshold and method-sensitive; no stable primary conclusion.
LOS endpoint excursion	χ^2^(4) = 1.362, *p* = 0.851	χ^2^(3) = 13.508, *p* = 0.004	χ^2^(12) = 65.304, *p* < 0.001	Condition and interaction effects retained in sensitivity analyses.
LOS maximum excursion	χ^2^(4) = 0.913, *p* = 0.923	χ^2^(3) = 22.718, *p* < 0.001	χ^2^(12) = 65.737, *p* < 0.001	Condition and interaction effects retained in sensitivity analyses.
LOS directional control	χ^2^(4) = 13.025, *p* = 0.011	χ^2^(3) = 15.204, *p* = 0.002	χ^2^(12) = 65.947, *p* < 0.001	Condition and interaction effects retained; no IQR outliers.

Note. Values are joint Wald χ^2^ tests from mixed-effects models containing participant-specific random intercepts and random linear condition slopes. Fixed auditory condition was modeled categorically. Complete coefficient estimates, model-based confidence intervals, post hoc contrasts, and sensitivity analyses are reported in Supplementary File S1.

**Table 2:** CTSIB composite score by sport type and auditory condition.

Sport	Routine	Low (+10 dB)	Moderate (+20 dB)	High (+30 dB)
Volleyball	67.83 ± 6.40	67.83 ± 7.17	71.83 ± 11.48	67.00 ± 4.47
Football	70.75 ± 10.04	67.50 ± 9.78	66.12 ± 12.06	67.25 ± 8.75
Swimming	69.78 ± 8.29	68.33 ± 10.63	68.44 ± 13.11	68.33 ± 12.33
Combat sports	66.64 ± 8.88	64.09 ± 13.00	65.64 ± 13.82	64.55 ± 14.89
Tennis	66.07 ± 11.52	51.64 ± 12.15	49.93 ± 11.73	43.71 ± 12.21

**Table 3:** CTSIB somatosensory score by sport type and auditory condition.

Sport	Routine	Low (+10 dB)	Moderate (+20 dB)	High (+30 dB)
Volleyball	91.33 ± 9.93	90.00 ± 10.66	88.00 ± 9.10	85.00 ± 6.72
Football	94.38 ± 9.83	90.62 ± 9.05	89.88 ± 8.25	87.62 ± 7.56
Swimming	94.67 ± 6.04	88.89 ± 9.23	90.89 ± 8.81	89.78 ± 9.24
Combat sports	92.64 ± 8.69	90.55 ± 9.49	90.36 ± 9.58	88.36 ± 9.91
Tennis	97.71 ± 4.32	84.57 ± 6.79	82.86 ± 7.61	76.86 ± 5.40

**Table 4:** CTSIB Visual Score by sport type and auditory condition.

Sport	Routine	Low (+10 dB)	Moderate (+20 dB)	High (+30 dB)
Volleyball	78.83 ± 17.34	78.50 ± 16.37	73.33 ± 11.98	73.33 ± 12.80
Football	78.38 ± 10.51	80.00 ± 8.58	78.50 ± 8.82	78.75 ± 9.97
Swimming	73.44 ± 22.34	72.56 ± 22.67	72.56 ± 21.33	73.11 ± 18.25
Combat sports	62.55 ± 23.44	64.18 ± 25.33	65.45 ± 27.06	67.09 ± 26.71
Tennis	47.43 ± 17.39	33.50 ± 17.43	34.36 ± 16.25	32.64 ± 17.43

**Table 5:** CTSIB vestibular score by sport type and auditory condition.

Sport	Routine	Low (+10 dB)	Moderate (+20 dB)	High (+30 dB)
Volleyball	79.67 ± 7.39	78.17 ± 7.44	77.67 ± 7.42	74.83 ± 7.49
Football	83.12 ± 9.14	82.88 ± 8.76	83.12 ± 8.49	80.88 ± 11.53
Swimming	76.44 ± 17.58	72.44 ± 20.34	72.56 ± 21.41	73.44 ± 18.70
Combat sports	74.09 ± 15.74	71.55 ± 20.35	73.73 ± 21.56	73.27 ± 22.86
Tennis	66.79 ± 14.45	52.36 ± 16.21	53.57 ± 17.62	49.21 ± 19.25

**Table 6:** CTSIB visual preference score by sport type and auditory condition.

Sport	Routine	Low (+10 dB)	Moderate (+20 dB)	High (+30 dB)
Volleyball	88.00 ± 6.42	87.33 ± 7.17	84.83 ± 6.74	86.83 ± 7.70
Football	92.25 ± 8.84	91.25 ± 7.65	89.75 ± 6.56	90.75 ± 7.27
Swimming	95.56 ± 4.22	94.78 ± 4.97	93.78 ± 7.05	95.11 ± 4.78
Combat sports	91.55 ± 6.17	90.45 ± 6.06	89.36 ± 5.61	91.09 ± 7.29
Tennis	92.07 ± 6.15	81.50 ± 6.44	81.07 ± 6.46	74.64 ± 10.45

**Table 7:** LOS reaction time (s) by sport type and auditory condition (mean ± SD).

Sport	Routine	Low (+10 dB)	Moderate (+20 dB)	High (+30 dB)
Volleyball	0.88 ± 0.16	0.79 ± 0.13	0.69 ± 0.19	0.54 ± 0.23
Football	0.92 ± 0.35	0.92 ± 0.35	0.91 ± 0.36	0.94 ± 0.36
Swimming	0.72 ± 0.22	0.75 ± 0.23	0.81 ± 0.26	0.87 ± 0.30
Combat sports	0.79 ± 0.26	0.78 ± 0.27	0.79 ± 0.26	0.78 ± 0.29
Tennis	0.92 ± 0.28	1.04 ± 0.36	1.11 ± 0.43	1.36 ± 0.85

Note. The primary analysis retained all observations. Values flagged by the 1.5 × IQR rule were retained in the primary analysis and excluded only in the sensitivity analysis reported in Supplementary File S1.

**Table 8:** LOS movement velocity by sport type and auditory condition (mean ± SD).

Sport	Routine	Low (+10 dB)	Moderate (+20 dB)	High (+30 dB)
Volleyball	3.14 ± 1.31	3.24 ± 1.27	3.17 ± 1.07	3.24 ± 1.35
Football	2.69 ± 0.77	2.71 ± 0.80	2.82 ± 0.99	2.65 ± 0.83
Swimming	2.82 ± 0.49	2.72 ± 0.51	2.64 ± 0.57	2.41 ± 0.56
Combat sports	3.05 ± 1.18	3.05 ± 1.16	2.91 ± 1.14	2.96 ± 1.11
Tennis	2.91 ± 0.63	2.95 ± 0.80	2.83 ± 0.85	2.65 ± 0.78

**Table 9:** LOS endpoint excursion by sport type and auditory condition (mean ± SD).

Sport	Routine	Low (+10 dB)	Moderate (+20 dB)	High (+30 dB)
Volleyball	54.66 ± 11.49	56.19 ± 11.56	55.71 ± 11.63	61.27 ± 10.47
Football	52.19 ± 9.47	52.93 ± 10.30	52.91 ± 12.00	48.44 ± 9.02
Swimming	51.03 ± 15.63	50.07 ± 15.01	47.39 ± 14.40	44.62 ± 14.80
Combat sports	50.72 ± 18.72	51.41 ± 18.62	51.51 ± 19.03	51.51 ± 18.75
Tennis	56.08 ± 14.09	53.26 ± 13.46	50.18 ± 12.83	46.28 ± 12.22

**Table 10:** LOS maximum excursion by sport type and auditory condition (mean ± SD).

Sport	Routine	Low (+10 dB)	Moderate (+20 dB)	High (+30 dB)
Volleyball	80.24 ± 21.58	79.67 ± 17.70	82.31 ± 17.64	84.23 ± 12.77
Football	79.16 ± 15.84	79.63 ± 15.94	78.83 ± 16.56	77.27 ± 17.43
Swimming	82.12 ± 14.27	80.13 ± 14.50	76.55 ± 14.46	68.33 ± 20.97
Combat sports	76.77 ± 20.54	77.31 ± 20.61	76.18 ± 20.46	77.82 ± 20.65
Tennis	82.33 ± 9.89	77.96 ± 8.99	72.73 ± 8.75	65.30 ± 7.70

**Table 11:** LOS directional control by sport type and auditory condition (mean ± SD).

Sport	Routine	Low (+10 dB)	Moderate (+20 dB)	High (+30 dB)
Volleyball	82.99 ± 11.16	85.59 ± 8.29	86.22 ± 5.86	89.86 ± 4.36
Football	75.89 ± 15.44	75.66 ± 15.76	74.97 ± 16.73	75.04 ± 17.58
Swimming	73.42 ± 13.51	72.21 ± 13.47	69.47 ± 13.96	64.69 ± 15.83
Combat sports	72.65 ± 12.86	73.38 ± 12.54	72.60 ± 13.21	72.54 ± 14.67
Tennis	71.36 ± 11.29	66.66 ± 11.73	63.15 ± 12.46	56.65 ± 12.15

Note. No directional-control observation was flagged by the 1.5 × IQR rule. One tennis high-condition observation was missing.

## Data Availability

The de-identified participant-level condition dataset and complete statistical reanalysis are provided in Supplementary File S1. The available dataset contains condition-level outcome summaries rather than individual trial-level recordings. Any additional non-public study documentation may be requested from the corresponding author, subject to institutional and ethical requirements.

## Supplementary Materials

The following supporting information can be downloaded at https://www.mdpi.com/article/10.3390/brainsci16080772/s1; Supplementary File S1 contains the de-identified participant-level condition dataset, cell-level descriptive statistics and 95% confidence intervals, complete mixed-effects-model coefficients, omnibus tests, estimated marginal means, Bonferroni-adjusted contrasts, model-structure comparisons, diagnostic statistics, IQR outlier flags, and robust sensitivity analyses.

## Abbreviations

The following abbreviations are used in this manuscript:

CTSIB	Clinical Test of Sensory Interaction in Balance
LOS	Limits of Stability
SPL	Sound pressure level
VR	Virtual reality
WNSS	Weinstein Noise Sensitivity Scale
